# Transport and Association of Ions in Lithium Battery Electrolytes Based on Glycol Ether Mixed with Halogen-Free Orthoborate Ionic Liquid

**DOI:** 10.1038/s41598-017-16597-7

**Published:** 2017-11-27

**Authors:** Faiz Ullah Shah, Oleg I. Gnezdilov, Rashi Gusain, Andrei Filippov

**Affiliations:** 1 Chemistry of Interfaces, Luleå University of Technology, Luleå, SE-97187 Sweden; 2 Institute of Physics, Kazan Federal University, 420008 Kazan, Russia

## Abstract

Ion transport behaviour of halogen-free hybrid electrolytes for lithium-ion batteries based on phosphonium bis(salicylato)borate [P_4,4,4,8_][BScB] ionic liquid mixed with diethylene glycol dibutyl ether (DEGDBE) is investigated. The Li[BScB] salt is dissolved at different concentrations in the range from 0.15 mol kg^−1^ to 1.0 mol kg^−1^ in a mixture of [P_4,4,4,8_][BScB] and DEGDBE in 1:5 molar ratio. The ion transport properties of the resulting electrolytes are investigated using viscosity, electrical impedance spectroscopy and pulsed-Field Gradient (PFG) NMR. The apparent transfer numbers of ions are calculated from the diffusion coefficients measured by using PFG NMR. PFG NMR data suggested ion association upon addition of Li salt to the [P_4,4,4,8_][BScB] in DEGDBE solution. This is further confirmed by liquid state ^7^Li and ^11^B NMR, and FTIR spectroscopic techniques, which suggest strong interactions between the lithium cation and oxygen atoms of the [BScB]^−^ anion in the hybrid electrolytes.

## Introduction

The continuous emission of CO_2_ into the atmosphere is thought to have catastrophic and irreversible effects on our planet such as rising of sea levels, acidification of oceans, polar melts, droughts, flood and global warning^1^. In this context, efficient electrical energy storage devices are highly desired in order to keep control on CO_2_ emissions into the atmosphere. This is one of the main reasons for the growing interest in electrical energy storage devices for transportation technologies. In this context, lithium-ion batteries are attracting attentions of researchers due to their high energy density, acceptable cycle life, no memory effect and low self-discharging^2^. An electrolyte is a key component of lithium-ion batteries acting as an ionic conductor and electronic insulator. A desirable electrolyte should have the following properties^3^: (1) wide electrochemical window; (2) high ionic conductivity; (3) high chemical and thermal stability; (4) chemical intertness to other components of the cell such as electrodes, separator and the cell packing materials; (5) safe, non-toxic, economical and recyclable.

Extensive research efforts are being made to identify electrolytes that can perfectly meet the requirements of lithium-ion batteries. Despite the continuous research efforts, development of safe and efficient electrolytes remains a challenge. Ionic liquids (ILs) possess a combination of physicochemical properties such as high ionic conductivity, high chemical and electrochemical stability, non-flammability, non-volatility, high thermal stability, and wide liquid range^4,5^. ILs are salts composed of cations and anions that are liquids at or below 100 °C^6^. ILs are mixed with an appropriate lithium salt to make electrolyte for lithium-ion batteries. The unusual properties of IL electrolytes make them attractive alternatives compared to the conventional organic electrolytes. However, there are a number of drawbacks of IL electrolytes. For example, the high viscosity and cost of ILs need to be reduced in order to replace organic electrolytes with IL electrolytes in lithium-ion batteries^7^. Mixing ILs with organic solvents is one of the best options to not only reduce the high viscosity and cost of ILs but also improve the transport and electrochemical properties of electrolytes^8–10^. In addition, the conventional ionic liquid electrolytes contain anions with halogen atoms such as BF_4_^−^, PF_6_^−^, etc. Such anions are prone to hydrolysis in moisture conditions and thus produce toxic and corrosive products limiting their applicability in batteries. Therefore, halogen-free IL electrolytes are necessary for high performance of the batteries and enhanced safety^11,12^.

Orthoborate-based salts are believed to be more efficient than the conventional salt such as LiPF_6_ because they offer several advantages such as good thermal stability, high compatibility with cathode materials, no erosion of manganese and iron cathode materials, and are also halogen-free and non-toxic^13,14^. A variety of orthoborate-based lithium salts and their derivatives such as lithium bis[1,2-benzenediolato(2-)-O,O′]borate Li[BBB]^15^, lithium bis[2,3-naphtha-lene-diolato(2-)-O,O′]borate Li[BNB]^16^, lithium bis[2,2-biphenyldiolato(2-)-O,O′]borate Li[BBPB]^17^, lithium bis[croconato]borate Li[BCB]^18^ and lithium bis(salicylato)borate Li[BScB]^19,20^ have been studied in different organic solvents as electrolytes for lithium-ion batteries. Some of the commonly used organic solvents for orthoborate salts are ethylene carbonate (EC), propylene carbonate (PC), dimethyl carbonate (DMC), Diethyl carbonate (DEC), Ethyl sulfite (ES), Dimethyl sulfite (DMS), Diethyl sulfite (DES), gamma-butyrolactone, vinylene carbonate^21–24^. The main reason for the high ionic conductivity, wide electrochemical stability window and high thermal stability of orthoborate anions is their charge delocalization. Particularly, the aromatic orthoborate anions, also known as Hückel anions, are potential electrolytes for batteries as compared with the conventional salts^25,26^.A variety of lithium borate salts and their potential applications in high performance lithium batteries have been recently reviewed by Liu *et al* ^27^.

An appropriate organic solvent is critically important not only for the dissolution of lithium orthoborate salt but also for oxidative stability, thermally stability, satisfactory conductivity, cell performance and life cycle^28^.Ether based organic solvents (also known as glymes) are gaining attentions in lithium ion batteries due to a number of advantages such as low viscosity, high thermal stability and safety^29^^,^^30^. Recently, a number of studies have demonstrated that mixtures of ionic liquids and glymes possess excellent electrolyte properties and are promising solvents for lithium-ion batteries^31–35^.

In this paper, we explore the ion transport mechanism of ternary mixture of [P_4,4,4,8_][BScB] IL, diethylene glycol dibutyl ether (DEGDBE) and a Li[BScB] salt. DEGDBE has a flash point at 118 °C and high miscibility with [P_4,4,4,8_][BScB] IL. To the best of our knowledge, this is the first study on investigation of ion transport mechanism of orthoborate-based ionic liquid hybrid electrolytes. The Li[BScB] salt is dissolved in a mixture of [P_4,4,4,8_][BScB] IL and DEGDBE with different concentrations in the range from 0.15 mol kg^−1^ to 1.0 mol kg^−1^. Electrical impedance spectroscopy (EIS) and PFG NMR spectroscopy (NMR diffusometry) are employed to understand the ion transport mechanism in the ternary mixtures. The physical and transport properties are investigated using ionic conductivity, viscosity, thermal analysis, multinuclear ^13^C,^31^P, ^11^B and ^7^Li NMR spectroscopy, FTIR spectroscopy and NMR diffusometry.

## Experimental Section

### Materials

**Figure 1:**
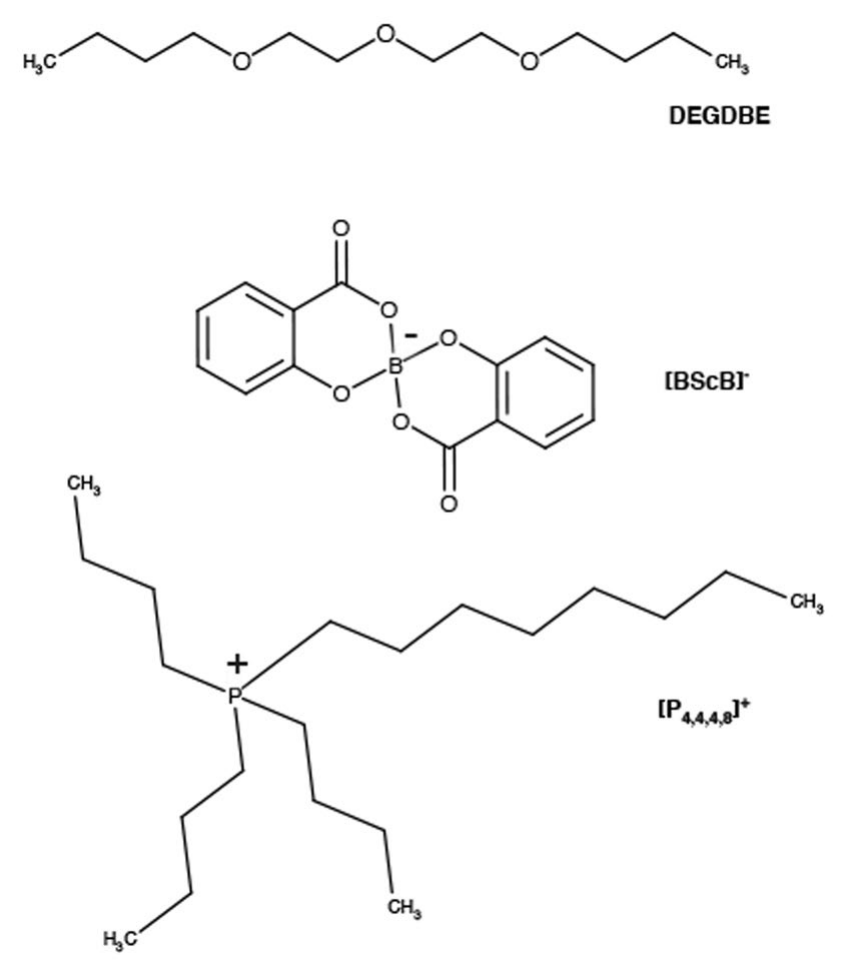
Structures and abbreviations of diethylene glycol dibutyl ether (DEGDBE) and tributyloctylphosphonium [P_4,4,4,8_]^+^ cation and bis(salicylato)borate anion [BScB]^−^ of the ionic liquid.

Ionic liquid [P_4,4,4,8_][BScB] and Li[BScB] salt were synthesized as described in our previous publication^36^. The structure and purity of the products were confirmed by using ^1^H,^13^C, ^31^P and ^11^B NMR spectroscopy. The samples were dried in a vacuum oven at 60 °C for more than 2 days before performing the experiments. As recently reported, the maximum solubility of Li[BScB] salt in [P_4,4,4,8_][BScB] IL is 0.418 mol kg^−1^ (20 mol%)^37^. In this work, the electrolytes were prepared by mixing diethylene glycol dibutyl ether (DEGDBE) and [P_4,4,4,8_][BScB] IL in 5:1 molar ratio. Li[BScB] salt was dissolved in the DEGDBE (Sigma-Aldrich, purity ≥ 99%) and ionic liquid mixture in various concentration ranging from 0.15 mol kg^−1^ to 1.0 mol kg^−1^. It is worth noting that the solubility of Li[BScB] salt is ca. 2.4 times higher in [P_4,4,4,8_][BScB] IL and DEGDBE mixture as compared with the neat [P_4,4,4,8_][BScB] IL. The chemical structures of DEGDBE and ionic components of [P_4,4,4,8_][BScB] IL are shown in Fig. 1.

The water content of the electrolytes was determined by Karl Fischer titration using 917 coulometer (Metrohm). The reported values are average of three independent measurements with standard deviation (SD). The water content of neat DEGDBE and [P_4,4,4,8_][BScB] IL were found to be 0.044 ± 0.001% and 0.039 ± 0.002%, respectively Table 1. The composition and water content of the hybrid electrolytes are shown in Table 1.

**Table 1:** Composition and water content of the hybrid electrolytes investigated in this study.

Li[BScB] concentration (mol kg^−1^)	Molar ratio			Water content (%) ± SD
	DEGDBE	[P_4,4,4,8_][BScB]	Li[BScB]	
0	5	1	0	0.163 ± 0.003
0.15	5	1	0.25	0.584 ± 0.013
0.30	5	1	0.5	0.614 ± 0.016
0.60	5	1	1.0	1.016 ± 0.040
0.90	5	1	1.5	1.325 ± 0.046
1.0	5	1	1.75	1.416 ± 0.054

### NMR Spectroscopy

The lithium salt and ionic liquid were characterized using Bruker Ascend Aeon WB 400 (Bruker BioSpin AG, Fällanden, Switzerland) NMR spectrometer. NMR spectra of the electrolytes were recorded with CDCl_3_ solvent as an external lock. The working frequency was 100.63 MHz for ^13^C, 162.01 MHz for ^31^P, 128.40 MHz for ^11^B and 155.56 MHz for ^7^Li. Data were processed using Bruker Topspin 3.5 software.

### NMR Diffusometry

NMR diffusion measurements were carried out on a Bruker Avance III (Bruker BioSpin AG, Fällanden, Switzerland) NMR spectrometer using PFG NMR probe Diff50 (Bruker) with a maximum amplitude of the magnetic field gradient pulse of 30 T m^−1^. The working frequencies for ^1^H and ^7^Li were 400.27 MHz and 155.56 MHz, respectively. For each NMR experiment, the sample was placed in a 5 mm glass tube and closed with a plastic stopper to avoid contact with air. The sample was equilibrated at a specific temperature for at least 30 min prior to measurements.

The detailed description of PFG NMR technique for measurement of molecular diffusion coefficients is reported earlier^38^. The primary information for the diffusion can be obtained from the diffusion decay (DD) of the NMR stimulated echo amplitude *A*. Diffusion decay of *A* for the stimulated echo pulse sequence in the case of simple non-associating molecular liquid can be described by the following equation^39^:

1 $$A(g,\delta ,{t}_{d})=A(0)\exp (-{\gamma }^{2}{g}^{2}{\delta }^{2}D{t}_{d})$$

where *A*(0) is a factor proportional to the proton content in the system and is dependent on the spin-lattice and spin-spin relaxation times, *γ* is the gyromagnetic ratio for a specific nucleus; *g* and *δ* are the amplitude and duration of the gradient pulse; *t*_*d*_ is a diffusion time; and *D* is the self-diffusion coefficient. In our experiments, *t*_*d*_ was in the range 4–100 ms for ^1^H diffusion, while *t*_*d*_ was in the range 200–700 ms for ^7^Li diffusion.

### Ionic Conductivity

The measurements of ionic conductivity of the electrolytes were carried out using Metrohm Autolab PGSTAT302N electrochemical workstation with FRA32M module for impedance measurements. A frequency range from 1 Hz to 1 MHz with an AC voltage amplitude of 50 mV_rms_ was used for the measurements. About 70 μL of the electrolyte sample was placed in a TSC70 closed cell (70 μL solvent) from RHD instruments. The experiments were performed in temperature range from −20 °C to 100 °C within an uncetainity of ± 0.1 °C. A two-electrode setup was used: the Pt-wire with a diameter of 0.25 mm as a working electrode and a 70 μL platinum crucible as a sample container and a counter electrode. Prior to each measurement, both the electrodes were polished with a Kemet diamond paste 0.25 μm. The cell constant (Kcell = 17.511 cm^−1^) was used for each experiment. The TSC70 closed cell was thermally equilibrated for at least 20 min before performing each experiment.

### Cyclic Voltammetry

Cyclic voltammetry (CV) experiments were performed using Autolab potentiostat PGSTAT302N (Metrohm) in a closed sample container in air. About 70 μL of the electrolyte was placed in a sealed RHD instruments Microcell HC. Experiments were carried out at 20 °C temperature using 0.1 M tetrabutylammonium hexafluorophosphate as a supporting electrolyte. A three electrode setup was used: Pt crucible worked as a counter electrode, glass sealed Pt wire acted as a working electrode and Ag/AgCl electrode was used as a reference electrode, to perform the experiments. Both the counter and working electrodes were polished with a Kemet diamond paste 0.25 μm prior to each measurement. All the CVs were recorded with a scan speed of 100 mV s^−1^. The potential of reference electrode was determined using ferrocene as an internal standard. The CV measurements are reported versus Li/Li^+^ redox couple reference potential (−3,04 V vs SHE). The values of potential against Li/Li^+^ were placed on reference scale by evaluating the formal potential of ferrocene (0.532 V vs SHE, 3.572 V vs Li/Li^+^).

### Infrared Spectroscopy

Fourier Transform Infrared (FTIR) spectra were recorded by applying a thin film of electrolyte on the KBr pellet using Bruker IFS 80 v vacuum fourier transform infrared spectrometer equipped with a deuterated triglycine sulphate (DTGS) detector. The experiments were carried out at room temperature (~22 °C) using the double side forward-backward acquisition mode. The total number of scans were 256 and signal-averaged at an optical resolution of 4 cm^−1^.

## Results and Discussion

**Figure 2:**
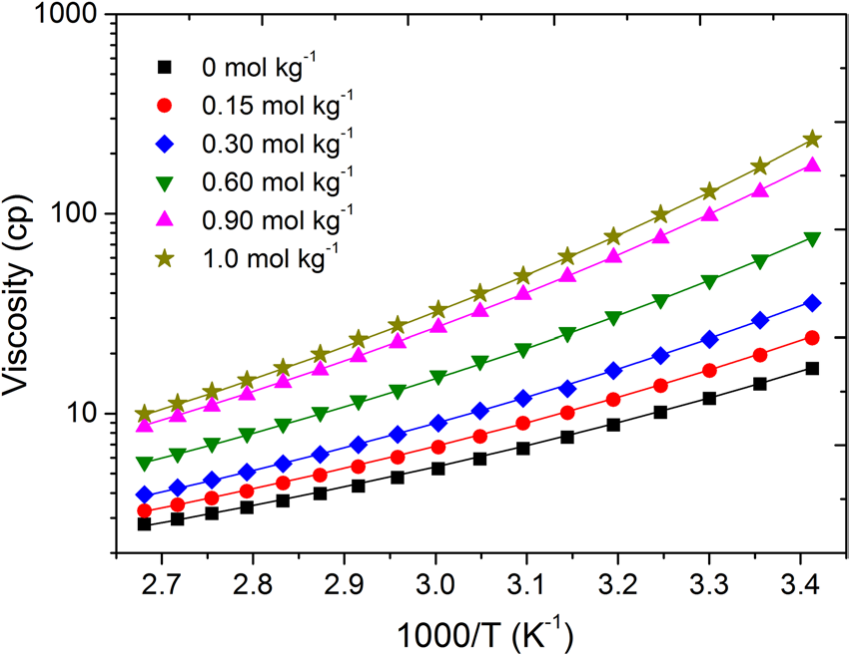
Dynamic viscosity of [P_4,4,4,8_][BScB] IL based hybrid electrolytes. Symbols indicate the experimentally measured values and solid lines present best the fittings using VFT equation (2) with parameters of Table 2.

The dynamic viscosity of [P_4,4,4,8_][BScB] IL based hybrid electrolytes with various concentrations of Li[BScB] salt is shown in Fig. 2. The viscosity of [P_4,4,4,8_][BScB] is significantly reduced when mixed with DEGDBE at 1:5 molar ratio. For example, the viscosity of the mixture of [P_4,4,4,8_][BScB] and DEGDBE (1:5 molar ratio) is 1.25 cP at 20 °C temperature, while the viscosity of neat [P_4,4,4,8_][BScB] IL is 1400 cP at the same temperature^36^.It is clearly seen that viscosity increases with increase in the concentration of lithium salt and decreases rapidly with increase in temperature. As it is observed earlier for other hybrid electrolytes, the viscosity values of [P_4,4,4,8_][BScB] IL based hybrid electrolytes follow Vogel-Fulcher-Tammann (VFT) behaviour as well^40^.

2 $$\eta ={\eta }_{0}^{\ast }\exp (\frac{B}{(T-{T}_{0})})$$

where ${\eta }_{0}^{\ast }$, *B*, and *T*_0_ are the fitting parameters: pre-exponential factor, a factor related to the activation energy and the ideal glass transition temperature, respectively. *T*_0_ indicates a temperature at which free volume and mobility is reduced to zero^41^. The fitting procedure was performed in two steps, as previously reported^37^. In the first step, ln(*η*) was plotted against 1/(*T*-*T*_0_) and selected *T*_0_ to have this dependence linear. The uncertainity is this step was ± 10 K. In the second step, the dependence was fitted by a linear regression to obtain the fitting parameters (${\eta }_{0}^{\ast }$, *B*). Activation energy for viscosity is related with *B* as *E*_*η*_ = *B*·*R*, where *R* is a gas constant. The VFT equation parameters and activation energies for viscosity data are tabulated in Table 2.

**Table 2:** VFT equation parameters and activation energies for viscosity data of [P_4,4,4,8_][BScB] IL based hybrid electrolytes.

Li[BScB] concentration (mol kg^−1^)	${\eta }_{0}^{\ast }$, cp	*B*	*T*_0_, K	*E*_*η*_, kJ/(mol·K)
0	0.165	570	170	4.7
0.15	0.145	630	170	5.2
0.30	0.123	700	170	5.8
0.60	0.105	810	170	6.7
0.90	0.085	940	170	7.8
1.0	0.075	990	170	8.2

**Figure 3:**
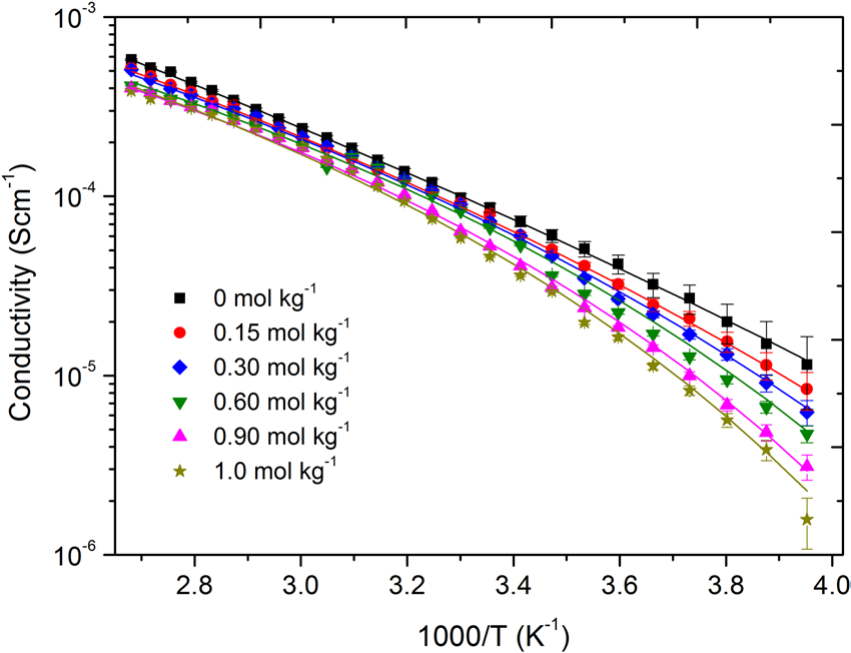
Ionic conductivity of [P_4,4,4,8_][BScB] IL based hybrid electrolytes and their best fittings using VFT equation. Symbols indicate the experimentally measured values and solid lines present the best fittings using VFT equation (3) with parameters of Table 3.

The ionic conductivity of the hybrid electrolyte with various concentration of Li[BScB] salt is shown in Fig. 3. Unlike viscosity, no significant change is observed in the ionic conductivity when [P_4,4,4,8_][BScB] is mixed with DEGDBE at 1:5 molar ratio. For example, the ionic conductivity of the mixture of [P_4,4,4,8_][BScB] and DEGDBE (1:5 molar ratio) mixture is found to be 7.3 × 10^−5^ S cm^−1^ and 5.8 × 10^−4^ S cm^−1^ at 20 °C and 100 °C temperatures, respectively. While the ionic conductivity obtained for neat [P_4,4,4,8_][BScB] IL is 3.3 × 10^−5^ S cm^−1^ at 20 °C and 1.1 × 10^−3^ S cm^−1^ 100 °C temperature^37^. The higher ionic conductivity of the neat [P_4,4,4,8_][BScB] IL at 100 °C temperature reveals efficient dissociation of ions at higher temperature. On the contrary, the ions remain together in the mixture of [P_4,4,4,8_][BScB] IL and DEGDBE even at higher temperature. The ionic conductivity decreases with the increase in the concentration of Li salt and it increases with increase in temperature for all the electrolytes studied. The addition of Li[BScB] salt causes an increase in viscosity and decrease in ionic conductivity of the electrolytes due to stronger coulombic interactions between the smaller lithium ions and the [BScB]^−^ anions as compared with the interactions between the larger phosphonium cations and the [BScB]^−^ anions^42,43^. The VFT equation is for ionic conductivity is^40^:

3 $$\sigma ={\sigma }_{0}^{\ast }\exp (\frac{-B}{(T-{T}_{0})})$$

where *σ*_0_^*^, *B*, and *T*_0_ are the fitting parameters. The fitting procedure was performed as in the case of viscosity. The temperature dependence of ionic conductivity for [P_4,4,4,8_][BScB] IL based hybrid electrolytes with different concentrations of Li[BScB] salt fit well to the VFT model over the entire temperature range studied. The VFT parameters for the ionic conductivity are tabulated in Table 3.

**Table 3:** VFT equation parameters and activation energies for ionic conductivity data of [P_4,4,4,8_][BScB] IL based hybrid electrolytes.

Li[BScB] concentration (mol kg^−1^)	*σ*_0_*	*B*	*T*_0_, K	*E*_*σ*_, kJ/(mol·K)
	S/cm			
0	0.15	1630	80	13.6
0.15	0.047	1150	120	9.6
0.30	0.027	940	140	7.8
0.60	0.014	740	160	6.2
0.90	0.012	690	170	5.7
1.0	0.015	730	170	6.1

**Figure 4:**
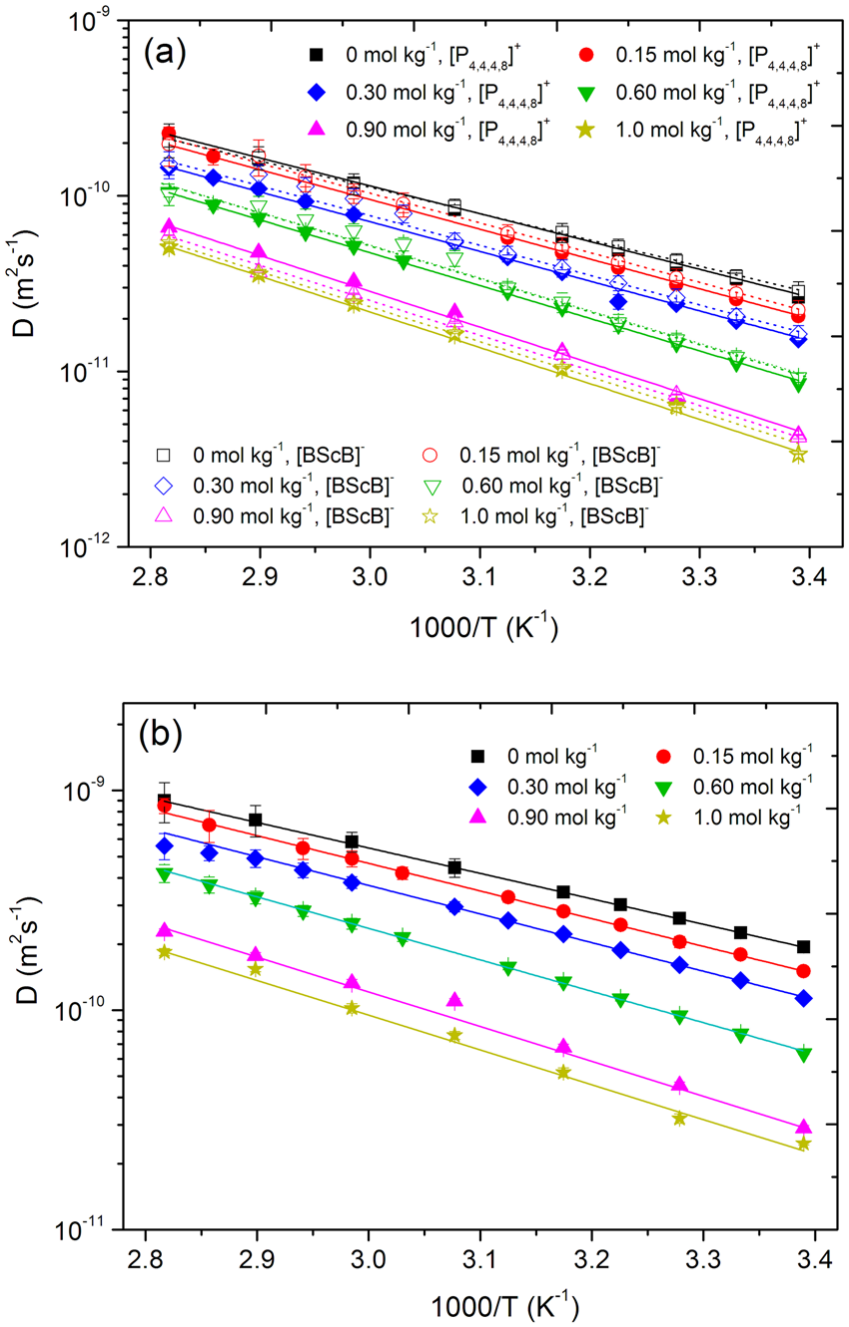
Arrhenius plot of diffusion coefficients of [P_4,4,4,8_]^+^ cation, [BScB]^−^ anion and DEGDBE in the ionic liquid based hybrid electrolytes and their best fittings using Arrhenius type (4). The error bars are contained within the size of the points.

**Figure 5:**
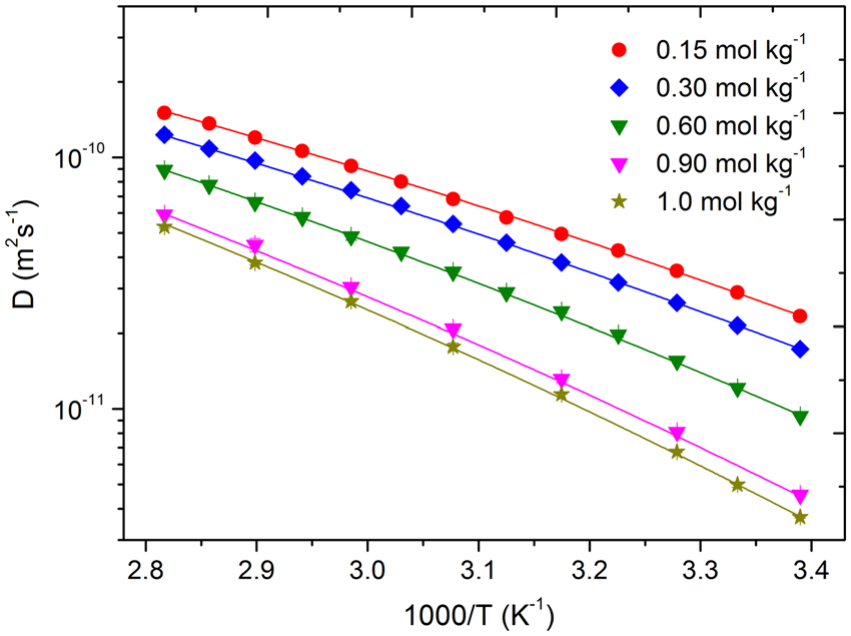
Arrhenius plot of diffusion coefficients of Li^+^ cation in the ionic liquid based hybrid electrolytes and their best fittings using VFT type equation (5). The error bars are contained within the size of the points.

**Figure 6:**
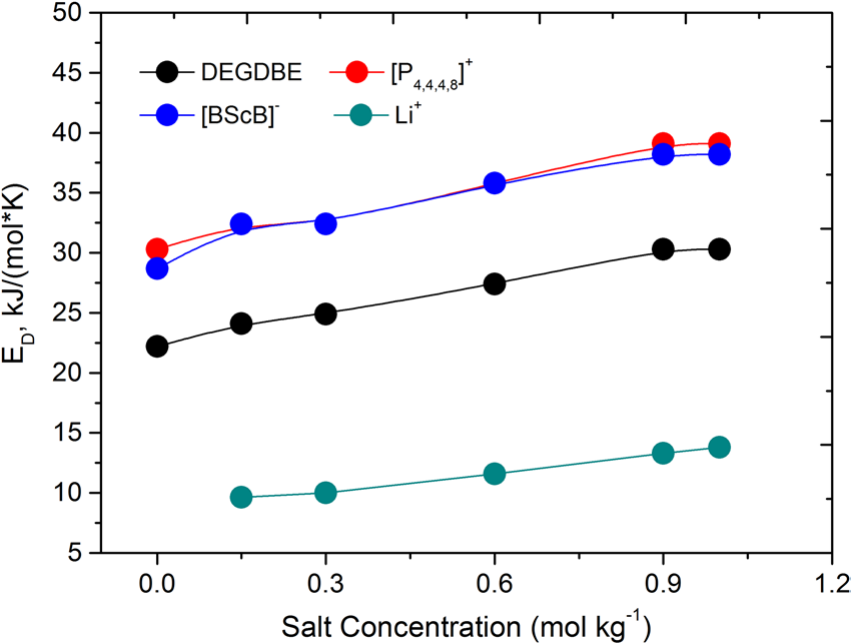
Apparent activation energy for diffusion of [P_4,4,4,8_]^+^ cation, [BScB]^−^ anion, DEGDBE and Li^+^ cation in the ionic liquid based hybrid electrolytes.

Diffusion decays for DEGDBE, [P_4,4,4,8_]^+^, [BScB]^−^, and Li^+^ were easily measured in the dynamic range of 2–3 decimal orders. Therefore, the achieved uncertainities in diffusion coefficients did not exceed 4.5%. This was estimated from the distribution of points in diffusion decays and also from the reproducibility values of diffusion coefficients in a consequent series of measurements. Figures 4 and 5 present the Arrhenius plots of diffusion coefficients of [P_4,4,4,8_]^+^ and [BScB]^−^ (Fig. 4a), DEGDBE (Fig. 4b) and Li^+^ (Fig. 5). The diffusion data demonstrate a monotonous increase in diffusivities of [P_4,4,4,8_]^+^, [BScB]^−^, DEGDBE and Li^+^ with increase in temperature. Diffusion coefficients of DEGDBE are higher by a factor of 4–6 as compared with the ions of the IL and Li^+^, while the diffusivities of ions in the IL and Li^+^ are comparable. As in the case of ionic conductivity, increase in the concentration of Li[BScB] lead to monotonous decrease in mobilities of all the four species present in the hybrid electrolyte system. Temperature dependences for [P_4,4,4,8_]^+^, [BScB]^−^ and DEGDBE can be described by Arrhenius dependence for diffusion:

4 $$D(T)={D}_{0}^{\ast }\cdot \exp (\frac{-{E}_{D}}{RT})$$

where *D*_0_^*^ is a parameter that is independent of temperature, *E*_*D*_ is the apparent molar activation energy of diffusion. Fitting parameters for diffusion of [P_4,4,4,8_]^+^, [BScB]^−^ and DEGDBE are presented in Table 4. Arrhenius function shows a linear dependence in Arrhenius coordinates. However, this is not the case for the diffusion of Li^+^ ions (Fig. 5). The universal form of temperature dependences of diffusion coefficients is the VFT equation in a form for diffusivity, which is equivalent to the Arrhenius dependence in the high-temperature limit^40,43^:

5 $$D={D}_{0}^{\ast }\exp (\frac{-B}{(T-{T}_{0})})$$

where *D*_0_^*^, *T*_0_, *B* are fitting parameters. Apparent energy of activation for diffusion is related with *B* as *E*_*D*_ = *B*·*R*. We have described *D*(*T*) in Fig. 5 by fitting *D*_0_^*^, *T*_0_ and *B*. Again, as in cases of viscosity and conductivity, the procedure of fitting was performed in two steps. The best fitting results are shown by solid lines in the Fig. 5 and the corresponding fitting parameters are tabulated in Table 4. Apparent energy of activation (*E*_*D*_) for diffusive motion of DEGDBE is always a factor ~1.3 lower as compared with the *E*_*D*_ of [P_4,4,4,8_]^+^ and [BScB]^−^. The difference in *E*_*D*_ observed for diffusion of [P_4,4,4,8_]^+^, [BScB]^−^ and Li^+^ is conditioned by the difference in *T*_0_. Increase in concentration of Li[BScB] in the electrolyte system leads to an increase in *E*_*D*_ of all four species. A graphical representation of *E*_*D*_ for all four species as a function of Li[BScB] salt is shown in Fig. 6 and the *E*_*D*_ values are tabulated in Table 4.

**Table 4:** The parameters of Arrhenius equation (4) and VFT equation (5), and activation energy for diffusion data for ionic liquid based hybrid electrolytes (Figs 4 and 5).

Li[BScB] concentration (mol kg^−1^)	Diffusing component	*D*_0_, m^2^/s	*B*	*T*_0_, K	*E*_*D*_, kJ/(mol·K)
0	DEGDBE	1.65 × 10^−6^			22.2
	[P_4,4,4,8_]^+^	6.5 × 10^−6^			30.3
	[BScB]^−^	3.5 × 10^−6^			28.7
0.15	DEGDBE	2.8 × 10^−6^			24.1
	[P_4,4,4,8_]^+^	11.5×10^−6^			32.4
	[BScB]−	12.5×10^−6^			32.4
	Li^+^	2.65×10^−8^	1160	130	9.64
0.3	DEGDBE	3.0 × 10^−6^			24.9
	[P_4,4,4,8_]^+^	8.6 × 10^−6^			32.4
	[BScB]^−^	9.3 × 10^−6^			32.4
	Li^+^	2.65 × 10^−8^	1210	130	10
0.6	DEGDBE	4.7×10^−6^			27.4
	[P_4,4,4,8_]^+^	19.0×10^−6^			35.8
	[BScB]^−^	21.0×10^−6^			35.8
	Li^+^	4.3×10^−8^	1390	130	11.6
0.90	DEGDBE	6.9 × 10^−6^			30.3
	[P_4,4,4,8_]^+^	3.8 × 10^−5^			39.1
	[BScB]^−^	2.5 × 10^−5^			38.2
	Li^+^	7.3 × 10^−8^	1600	130	13.3
1.0	DEGDBE	5.4 × 10^−6^			30.3
	[P_4,4,4,8_]^+^	2.9 × 10^−5^			39.1
	[BScB]^−^	2.3 × 10^−5^			38.2
	Li^+^	8.7 × 10^−8^	1660	130	13.8

The decrease in diffusion coefficients with an increase in Li[BScB] salt suggests that the added lithium ions increases the sizes of diffusing entities in the hybrid electrolyte. Indeed, the small Li^+^ ion diffuses with the same diffusion coefficient as the bulky [BScB]^−^ and [P_4,4,4,8_]^+^ ions. It means that the degree of dissociation of Li[BScB] is not very high and Li^+^ ions diffuse together with the [BScB]^−^ anion. This may happen if [P_4,4,4,8_]^+^, [BScB]^−^ and Li^+^ ions form associates, which present a diffusive entity in the hybrid electrolyte. It is worth noting that size of these associates increases as the concentration of Li[BScB] in the electrolyte system increases. The plausible explanation of this is stronger interactions between Li^+^ cation and [BScB]^−^ as compared with the interactions between [P_4,4,4,8_]^+^ cation and [BScB]^−^ anion. The increase in viscosity (Fig. 2) and decrease in ionic conductivity (Fig. 3) with the addition of lithium salts can be also explained by the increase in sizes of the diffusing entities in the hybrid electrolyte.

The apparent transference numbers of the ions were calculated from the self-diffusion coefficients using the following equation, as reported previously in a number of publications^44,45^.

6 $${t}_{i}=\frac{{x}_{i}{D}_{i}}{{{\rm{\Sigma }}}_{i}{x}_{i}{D}_{i}}$$

where ${t}_{i}$ is the apparent transference number, ${x}_{i}$ is the molar fraction of an ion and ${D}_{i}$ is the self-diffusion coefficient of an ion in m^2^s^−1^. The apparent transference numbers of Li^+^, [P_4,4,4,8_]^+^ and [BScB]^−^ ions for different concentrations and temperatures are presented in Table 5. As anticipated, the transference number of Li^+^ is significantly lower for electrolyte containing mixture of [P_4,4,4,8_][BScB] and DEGDBE as compared to [P_4,4,4,8_][BScB] based electrolyte without DEGDBE. For example, the transference number of Li^+^ in the hybrid electrolyte with lithium ion concentration of 0.30 mol kg^−1^ is 0.011 at 295 K, while it is 0.040 for [P_4,4,4,8_][BScB] based electrolyte without DEGDBE under similar experimental conditions^37^. The apparent transference numbers of Li^+^ ion are lower in electrolyte containing mixture of [P_4,4,4,8_][BScB] and DEGDBE as compared with [P_4,4,4,8_][BScB] based electrolyte, although the concentration of lithium ion is 2.5 fold higher in the former electrolyte. The lower transference numbers of Li^+^ ion suggest that the ions are moving together in the form of clusters. Even the apparent transference numbers of the [P_4,4,4,8_]^+^ cation and [BScB]^−^ anion are comparable with the [P_4,4,4,8_][BScB] based electrolyte without DEGDBE. It reveals that the addition of DEGDBE did not affect the transference numbers of ions, however, the viscosity of the resulting electrolytes is significantly reduced.

**Table 5:** Apparent transference numbers of individual ions at various temperatures for the ionic liquid based hybrid electrolytes.

Li[BScB] concentration (mol kg^−1^)	295 K			325 K			355 K		
	*t*_Li_	*t*_[P4,4,4,8]+_	*t*_[BScB]−_	*t*_Li_	*t*_[P4,4,4,8]+_	*t*_[BScB]−_	*t*_Li_	*t*_[P4,4,4,8]+_	*t*_[BScB]−_
0		0.482	0.518		0.490	0.510		0.508	0.492
0.15	0.005	0.474	0.521	0.005	0.478	0.518	0.004	0.532	0.465
0.30	0.011	0.472	0.517	0.010	0.484	0.506	0.008	0.479	0.523
0.60	0.021	0.461	0.518	0.018	0.428	0.555	0.017	0.488	0.495
0.90	0.015	0.476	0.492	0.031	0.501	0.469	0.028	0.496	0.475
1.0	0.015	0.462	0.500	0.038	0.458	0.505	0.035	0.450	0.514

There might be several reasons for the lower transference number of Li^+^ cation in the hybrid electrolytes. First, the Li^+^ cations could not be dissociated completely and form larger aggregates together with [P_4,4,4,8_]^+^ cations and [BScB]^−^ anions that diffuse slowly in the electrolytes. It reveals that there are strong interactions between the Li^+^ cation and [BScB]^−^ anion. Second, the sizes of [P_4,4,4,8_]^+^ cation and [BScB]^−^ anion are much larger than the typical ionic liquids comprising smaller cations such imidazolium and pyrrolidinium, and halogenated anions such as BF_4_, PF_6_ and NTf_2_. The slower diffusivity is due to their large sizes and since lithium atom is strongly interacting with the anion, the mobility of lithium is affected as well. Third, it is known that PFG NMR method underestimates the diffusion coefficients of charged species. For example, Martins *et al*. have compared the calculated transference numbers of lithium ion in ILs using NMR diffusometry and electrochemical methods. It was found that the values calculated from electrochemical method were 2–3 fold larger as compared with the values determined from NMR diffusometry^46^. NMR diffusometry takes into account all the species and aggregates, which might be larger than the small charged Li^+^ cation and thus, underestimates the diffusion coefficients of the charged species.

**Figure 7:**
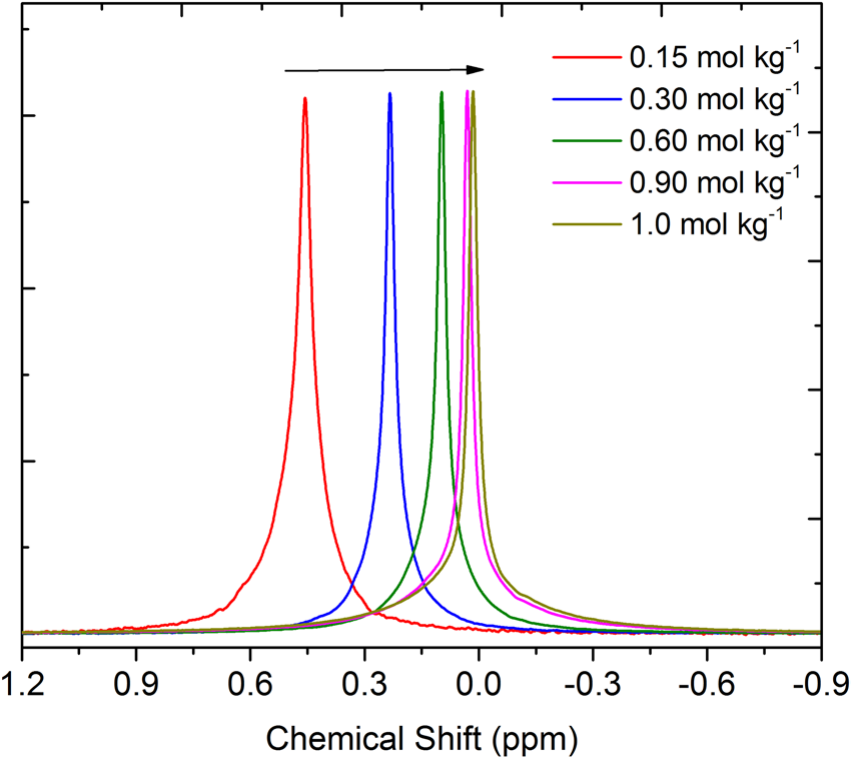
^7^Li NMR spectra of [P_4,4,4,8_][BScB] IL based hybrid electrolytes at 22 °C. 1 M LiCl was used as an external reference. Arrow indicates direction of increasing Li[BScB] salt concentration.

**Figure 8:**
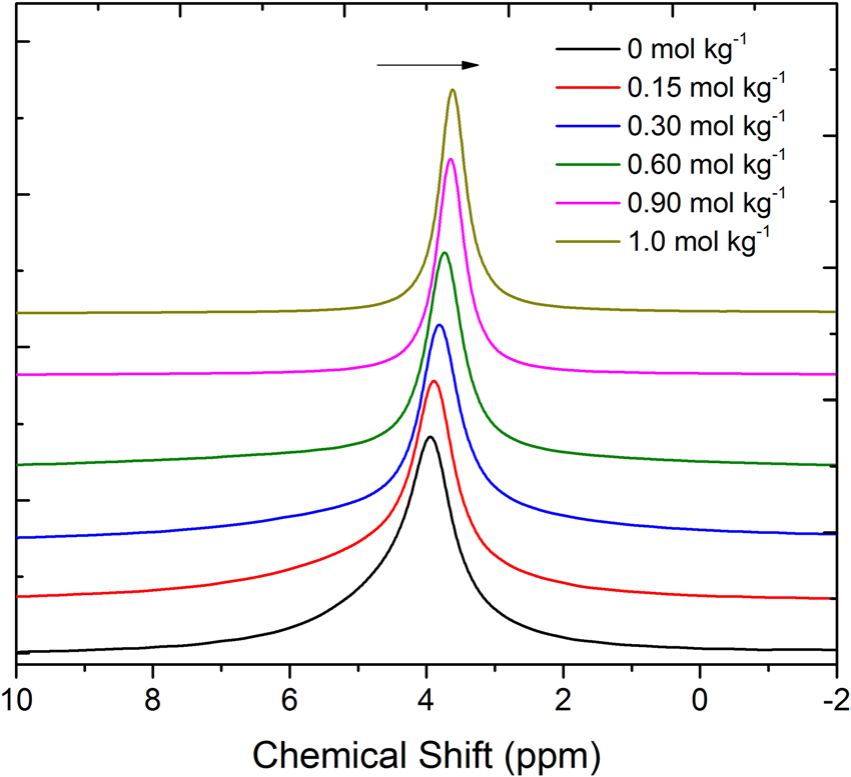
^11^B NMR spectra of [P_4,4,4,8_][BScB] IL based hybrid electrolytes at 22 °C. Arrow indicates direction of increasing Li[BScB] salt concentration.

In order to get deeper insights into the local environment, we performed ^7^Li, ^11^B, ^31^P and ^13^C NMR measurements of the electrolytes using CDCl_3_ as an external lock. As expected, significant changes in the chemical shifts of ^7^Li and ^11^B NMR spectra as a function of lithium salt concentration are observed (Figs 7 and 8). We did not observe any significant changes in the chemical shifts of the peaks for [P_4,4,4,8_]^+^ cation in ^31^P and ^13^C NMR spectra recorded under the same experimental conditions (see ESI). However, ^7^Li NMR peaks shift towards lower ppm values with increasing Li salt concentration in the electrolyte. This significant change in chemical shift suggests that the chemical environment of Li^+^ ions is changing with increasing concentration of Li salt in the electrolyte. It reveals that the Li^+^ ions are more shielded upon addition of Li salt due to the stronger interactions between Li^+^ ions and [BScB]^−^ anions. This is in agreement with the earlier findings by Yoon, *et al*. in electrolytes based on LiFSI dissolved in C_3_mpyrFSI ionic liquid; they have found that ^7^Li NMR spectra shift toward more negative values with increasing concentration of Li salt in the ionic liquid^47^. It is interesting to note that we observed similar changes towards lower ppm values in the ^11^B NMR spectra with increasing Li salt concentration as well (Fig. 8). This further confirms that addition of Li salt lead to stronger interactions between Li^+^ ions and [BScB]^−^ anions, which have shielded both lithium and boron nuclei.

**Figure 9:**
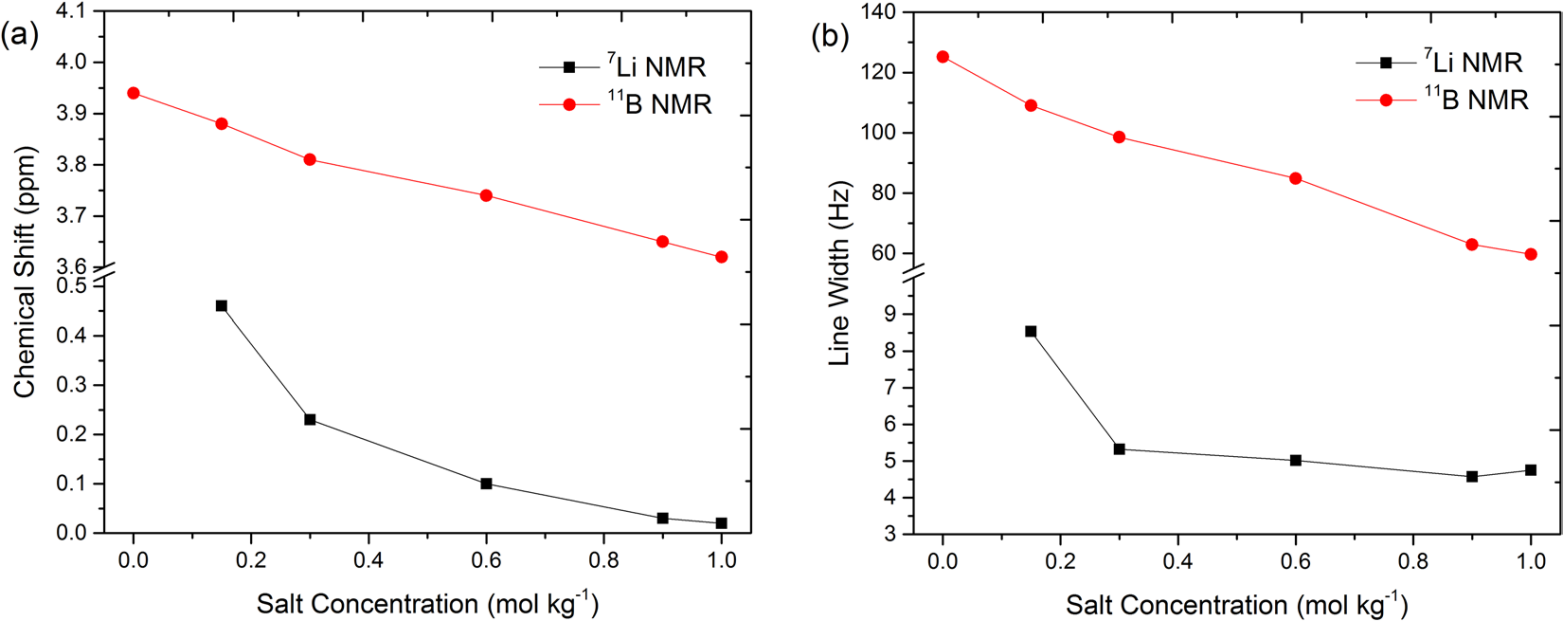
(**a**) Chemical shift and (**b**) line width of ^7^Li and ^11^B NMR spectra of [P_4,4,4,8_][BScB] IL based hybrid electrolytes at 22 °C.

A graphical representation of the changes in chemical shift and full width at maximum height (fwhm) as a function of Li salt concentration is shown in Fig. 9. A continuous shift towards lower ppm values is observed in the chemical shift of ^7^Li and ^11^B NMR spectra with addition of Li salt until it reaches the saturation point at 1.0 mol kg^−1^ concentration. Generally, the line width NMR peak increases with increasing Li salt concentrations and line width decreases with increasing temperature. Interestingly, we observed the opposite in the case of both ^7^Li and ^11^B NMR spectra of bis(salicylato)borate ionic liquid based hybrid electrolytes. The fwhm of ^11^B NMR spectra decreases continuously with increasing Li salt concentration, while in the case of ^7^Li NMR spectra a significant decrease is observed at 0.15 mol kg^−1^ concentration of Li salt and then decreases slightly upon addition of more Li salt. However, the fwhm increases again slightly at Li salt concentration above 0.90 mol kg^−1^. The changes in line width reveal variation in ion dynamics as a function of Li salt concentration. Hilder *et al*. have recently observed a decrease in the line width of ^23^Na NMR spectra with increase in concentration of Na salt and increase with increase in temperature^48^. They have suggested that the signal shape is heavily affected by the quadrupolar interactions. In our case, both ^7^Li and ^11^B have comparable quadrupolar moments and both nuclei have spin 3/2.

Another explanation of the observed changes in ^7^Li and ^11^B NMR chemical shift and line width with Li^+^ concentration is related to the specific structure of a complex formed by Li^+^, [P_4,4,4,8_]^+^ and [BScB]^−^ ions. With the increase in Li^+^ ion concentration, the concentration of [BScB]^−^ anions in the system remains unchanged but [P_4,4,4,8_]^+^ cations are regularly being replaced by Li^+^ ions. The alteration of chemical shifts of ^11^B and ^7^Li but no changes in chemical shifts of ^31^P with increasing lithium salt concentration demonstrates selective changes in packing of ions in the complex concerning mainly Li^+^ and [BScB]^−^. A decrease in line width is usually related with the decrease in transverse NMR relaxation rate (1/*T*_2_) and rotational correlation time of ions (τ_c_)^49^. Therefore, the decrease in line widths of ^11^B and ^7^Li NMR spectra is due to an increase in the rotational motion of Li^+^ and [BScB]^−^ ions because of replacement of the bulky [P_4,4,4,8_]^+^ cations with the small Li^+^ ions.

**Figure 10:**
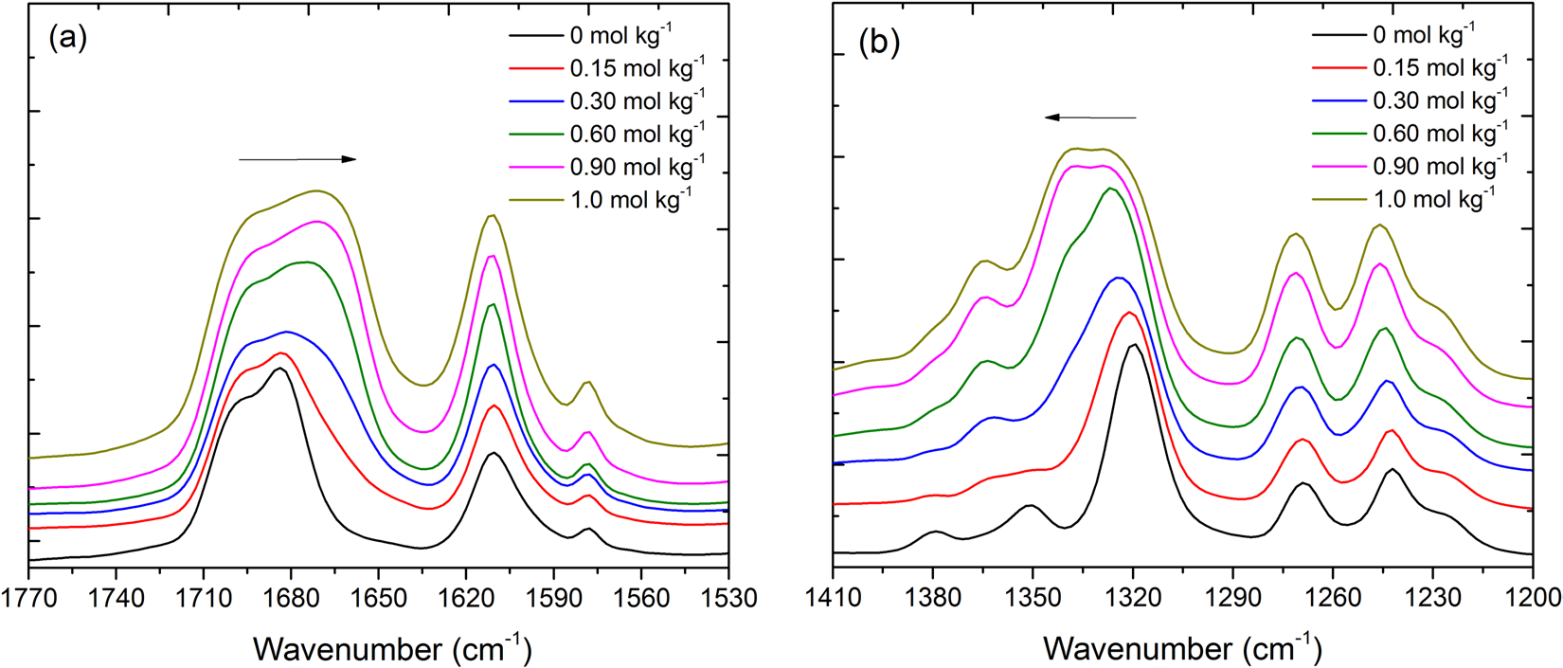
FTIR spectra of [P_4,4,4,8_][BScB] IL based hybrid electrolytes in the frequency range (**a**) from 1770 cm^−1^ to 1530 cm^−1^ and (**b**) from 1410 cm^−1^ to 1200 cm^−1^. Arrows indicate shift in wavenumber with increasing Li[BScB] salt concentrations.

FTIR spectroscopy was used to further investigate the interaction of Li^+^ ion with the [BScB]^−^ anion. The full FTIR spectra of bis(salicylato)borate ionic liquid based hybrid electrolytes with different concentrations of Li[BScB] salt are shown in ESI. Figure 10 shows selected regions of the FTIR spectra in the frequency range from 1770 cm^−1^ to 1530 cm^−1^ (Fig. 10a) and from 1410 cm^−1^ to 1200 cm^−1^ (Fig. 10b). The vibrations around 1700 cm^−1^ are assigned to C=O stretching and around 1610 cm^−1^ to the aromatic ring vibrations of [BScB]^−^ anion (Fig. 10a). As expected, the C=O band is shifted towards lower wavenumber values and peak broadening occurs upon addition of Li salt. This shift and broadening indicate the Li^+^ ion coordination with [BScB]^−^ anion. However, there is no change in the aromatic ring vibrations of [BScB]^−^ anion around 1610 cm^−1^ with increasing lithium salt concentration.

The band around 1320 cm^−1^ is attributed to the B-O stretching of [BScB]^−^ anion. Interestingly, this peak is also getting broader and shifted towards higher wavenumber when Li salt is added to the electrolyte. The peak broadness and shift reveal stronger interactions between the Li^+^ cations with the [BScB]^−^ via oxygen atoms in the anion. The FTIR data suggest that there are higher levels of ion pairing, which might lead to formation of aggregates at higher concentration of lithium salt in the electrolytes. This FTIR data support the findings of NMR spectroscopy.

The combined FTIR and NMR measurements suggested strong interactions between Li^+^ cations and [BScB]^−^ anions in ionic liquids based hybrid electrolytes. Recently, we have reported that the aromatic [BScB]^−^ anion may be coordinated through the delocalized electrons, one oxygen atom or two, and/or may be coordinated to a single Li^+^ cation or more^37^. Such interactions lead to ionic clustering at higher concentrations of Li salt in the ionic liquid. Forsyth *et al*. have previously observed similar ionic clustering at higher concentration of NaFSI salt in C_3_mPyr ionic liquid^50^. This work has demonstrated that ionic clustering occurs not only in neat ionic liquid based electrolytes but also in ionic liquid based hybrid electrolytes in the presence of organic solvent.

**Figure 11:**
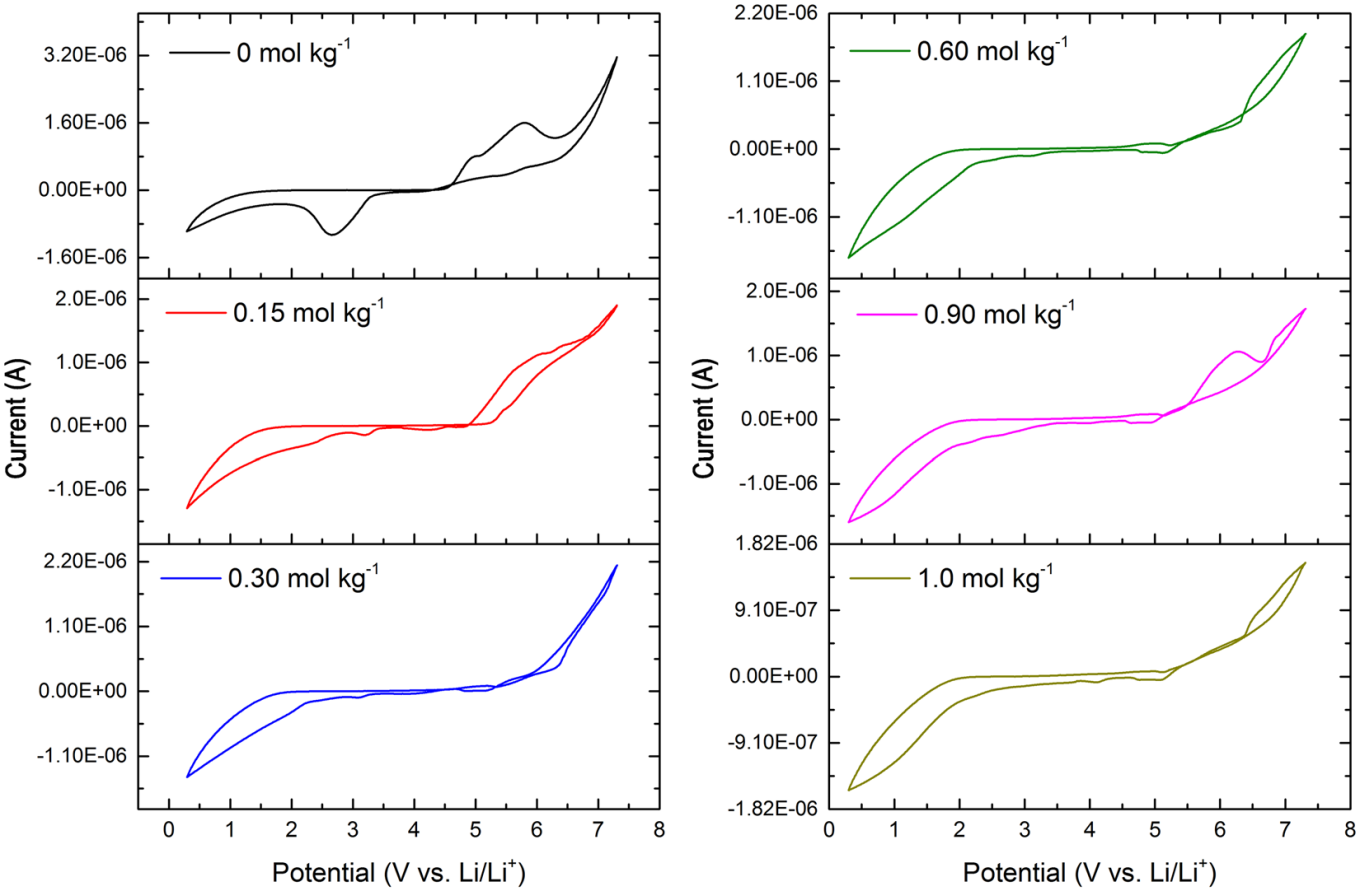
Cyclic voltammetry of [P_4,4,4,8_][BScB] IL based hybrid electrolytes at 20 °C temperature.

Figure 11 presents the cyclic voltammograms of [P_4,4,4,8_][BScB] IL based hybrid electrolytes at 20 °C temperature. It could evidently be seen that the concentration of Li salt has a remarkable effect on the observed electrochemical behavior of the electrolyte. Generally, the cathodic and anodic limits of the electrolytes are being set by the reduction of cation and oxidation of anion, respectively. The upper anodic limit for all electrolyte reaches ~6.0 V vs Li and this is the oxidation stability limit of [BScB]^−^ anion. The charge delocalization and π conjugated system in [BScB]^−^ anionic structure might be the reason of high oxidation potential of the electrolyte. The major variation in the stability of electrolyte is observed at cathodic reduction potential. It has been observed that the cathodic limit of [P_4,4,4,8_][BScB] IL and DEGDBE is slightly suppressed from 1.45 V to 1.55 V vs Li upon addition of 0.15 mol kg^−1^ Li[BScB] in the electrolyte. This further decreases to 1.9 V vs Li with the addition of 1.0 mol kg^−1^ of Li[BScB] salt. This reduction in cathodic limit might be due to the ion-ion interactions and association of ions in presence of Li salt, which probably leads to decrease in the availability of ions^42^. The electrochemical window of electrolytes was observed in the range of 4.5 to 5 V.

## Conclusions

The ion transport behaviour of halogen-free hybrid electrolytes based on [P_4,4,4,8_][BScB] IL mixed with DEGDBE in a 1:5 molar ratio was thoroughly investigated. A maximum solubility of Li[BScB] salt in a mixture [P_4,4,4,8_][BScB] and DEGDBE was 1.0 mol kg^−1^ at room temperature. The viscosity of the mixture was >1000 times lower as compared with the neat [P_4,4,4,8_][BScB] ionic liquid. However, no significant changes are observed in the ionic conductivity of the mixture as compared the neat [P_4,4,4,8_][BScB] ionic liquid. As expected, an increase in the ionic conductivity is observed with increase in temperature and a decrease is found with the addition of Li[BScB] salt to the mixture [P_4,4,4,8_][BScB] and DEGDBE. The PFG NMR data suggested that the ionic mobility is increasing at increase in temperature and decrease with increasing Li[BScB] concentrations. PFG NMR, liquid state ^7^Li and ^11^B NMR and FTIR spectroscopic techniques suggested strong interactions between the lithium cation and the [BScB]^−^ anion, which lead to ion association in the electrolytes. This ion association with addition of Li[BScB] salt to the mixture [P_4,4,4,8_][BScB] and DEGDBE decreases the mobility of ions as well as the solvent molecules.

## Electronic supplementary material

Supplementary Information
