# Supplementary material for: Transport and Association of Ions in Lithium Battery Electrolytes Based on Glycol Ether Mixed with Halogen-Free Orthoborate Ionic Liquid
Source: Sci Rep. 2017 Nov 27;7:16340. doi: 10.1038/s41598-017-16597-7 (PMC5703989; doi:10.1038/s41598-017-16597-7)
Supplement: Supplementary file 1 — Supplementary Information [file 41598_2017_16597_MOESM1_ESM.pdf]

Supporting Information

Transport and Association of Ions in Lithium Battery Electrolytes Based on Glycol Ether Mixed with Halogen-Free Orthoborate Ionic Liquid

Faiz Ullah Shah[*1], Oleg I. Gnezdilov[2], Rashi Gusain[1] and Andrei Filippov[*1,2]

[1]Chemistry of Interfaces, Luleå University of Technology, Luleå, SE-97187, Sweden

[2]Institute of Physics, Kazan Federal University, 420008 Kazan, Russia

[*]E-mail: faisha@ltu.se and andrei.filippov@ltu.se

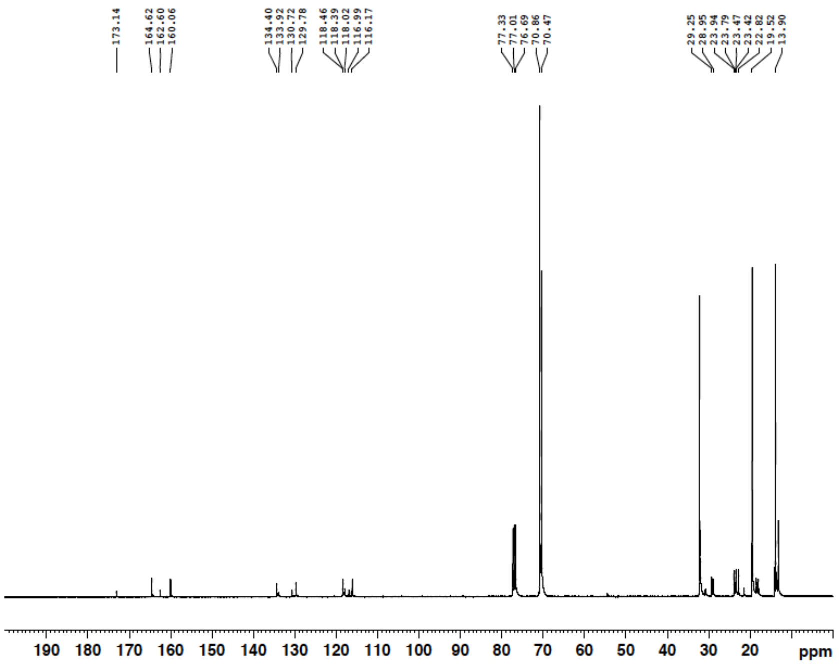

Figure ESI-1. ^{13}C NMR spectrum of mixture of DEGDBE and [$P_{4,4,4,8}$][BScB] ionic liquid with 0 mol kg^{-1} Li[BScB] salt.

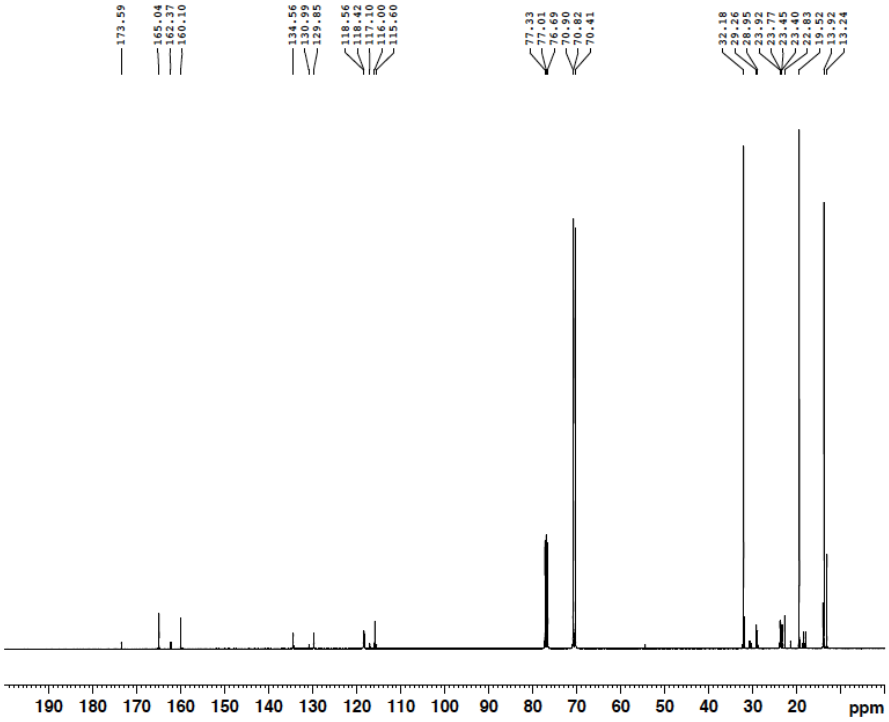

Figure ESI-2. ^{13}C NMR spectrum of mixture of DEGDBE and [$P_{4,4,4,8}$][BScB] ionic liquid with 0.15 mol kg^{-1} Li[BScB] salt.

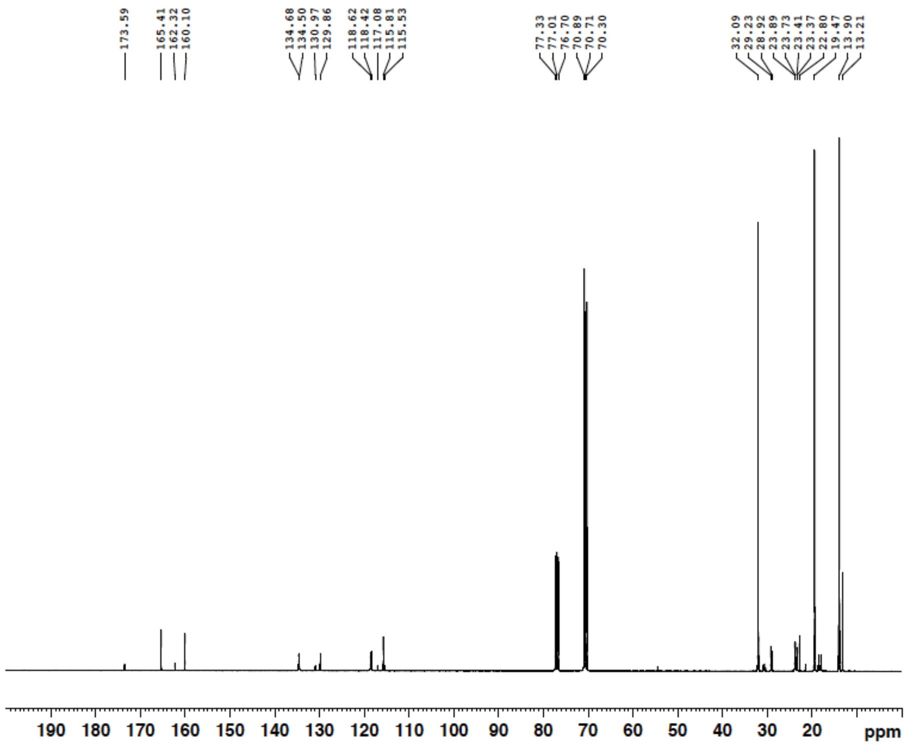

Figure ESI-3. ^{13}C NMR spectrum of mixture of DEGDBE and [$P_{4,4,4,8}$][BScB] ionic liquid with 0.30 mol kg^{-1} Li[BScB] salt.

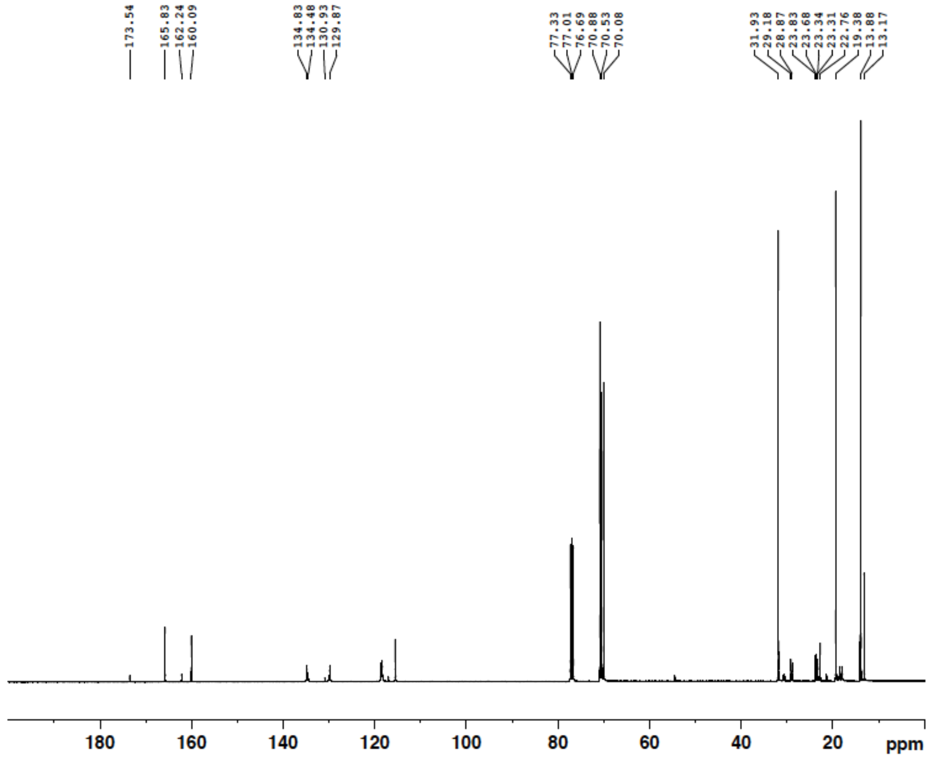

Figure ESI-4. ^{13}C NMR spectrum of mixture of DEGDBE and [$P_{4,4,4,8}$][BScB] ionic liquid with 0.60 mol kg^{-1} Li[BScB] salt.

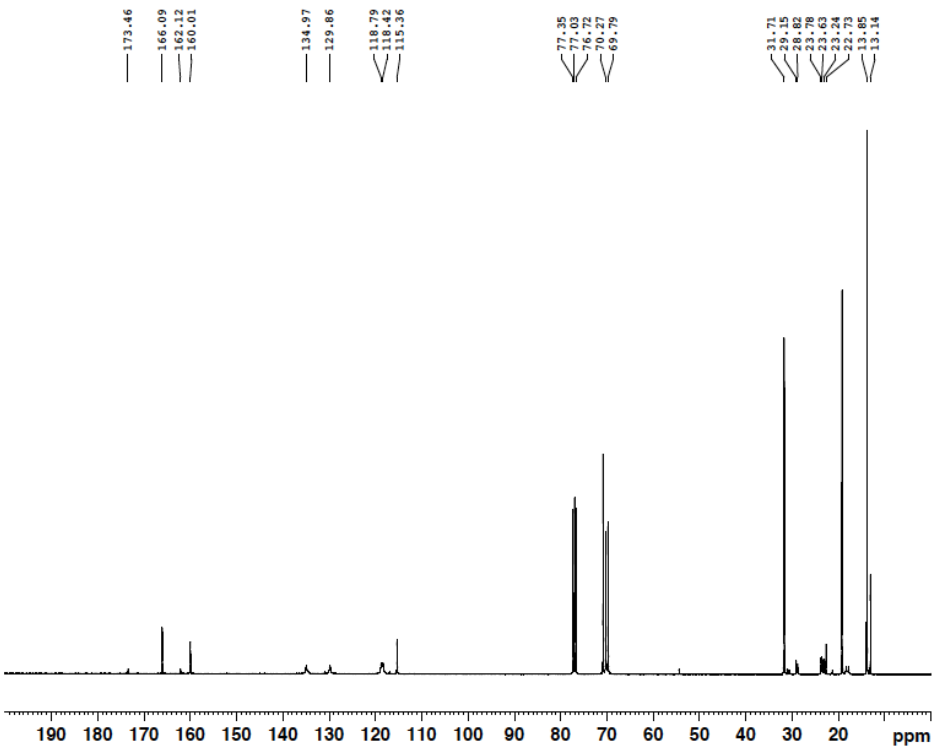

Figure ESI-5. ^{13}C NMR spectrum of mixture of DEGDBE and [$P_{4,4,4,8}$][BScB] ionic liquid with 1.0 mol kg^{-1} Li[BScB] salt.

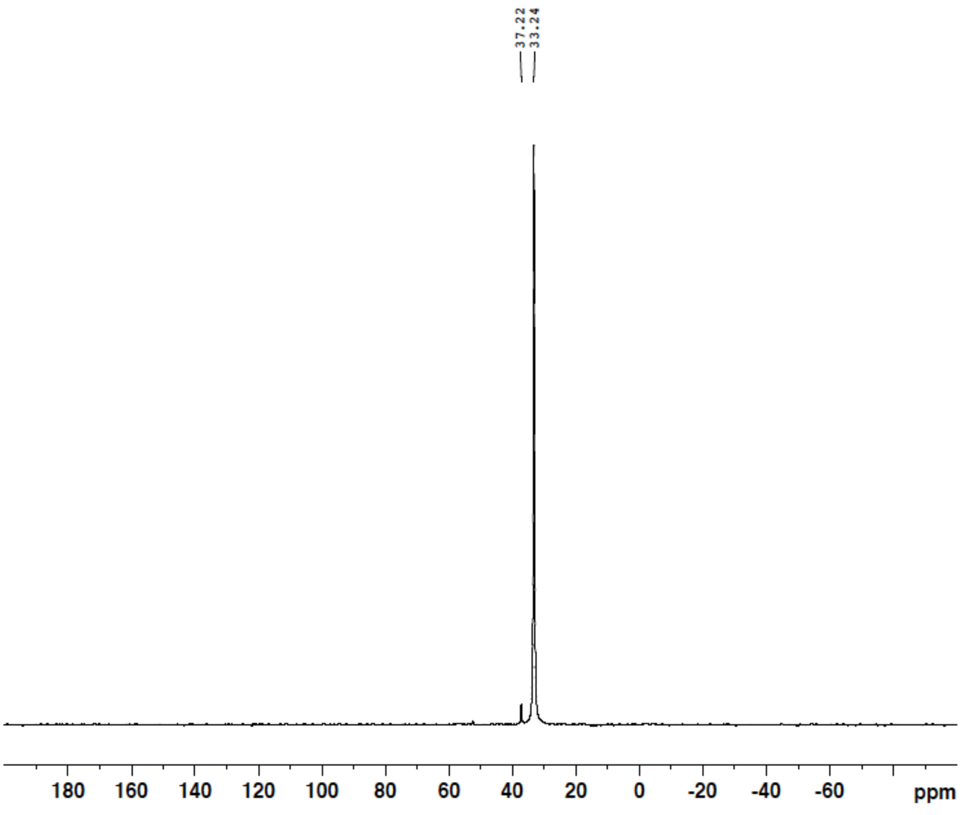

Figure ESI-6. ^{31}P NMR spectrum of mixture of DEGDBE and [$P_{4,4,4,8}$][BScB] ionic liquid with 0 mol kg^{-1} Li[BScB] salt.

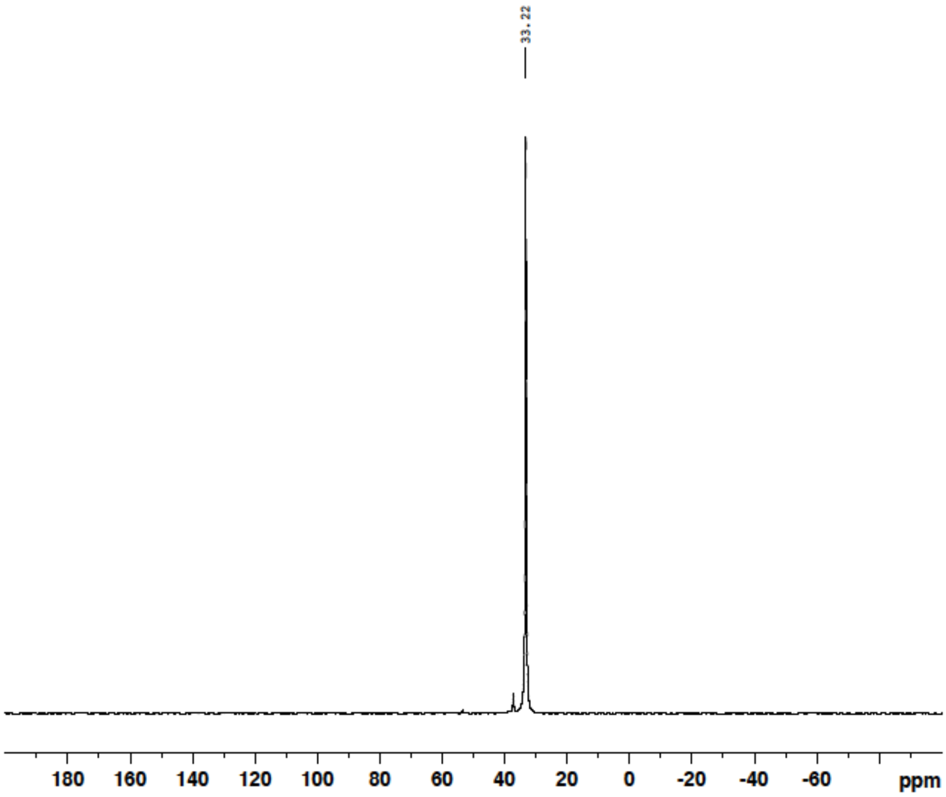

Figure ESI-6. ^{31}P NMR spectrum of mixture of DEGDBE and $[P_{4,4,4,8}]$[BScB] ionic liquid with 0.15 mol kg^{-1} Li[BScB] salt.

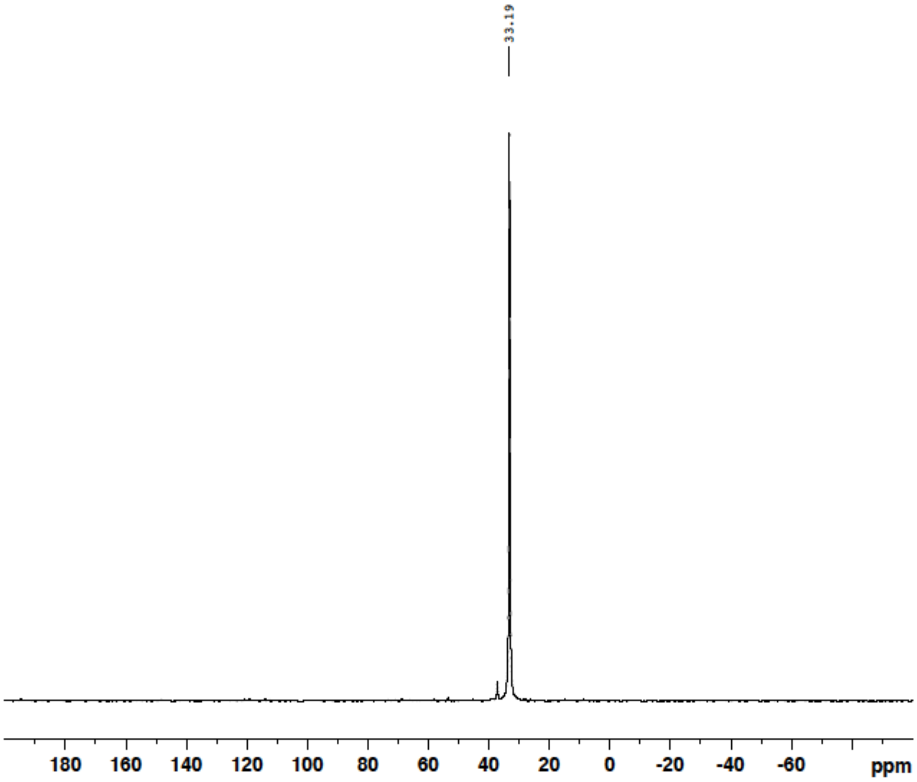

Figure ESI-7. ^{31}P NMR spectrum of mixture of DEGDBE and $[P_{4,4,4,8}]$[BScB] ionic liquid with 0.30 mol kg^{-1} Li[BScB] salt.

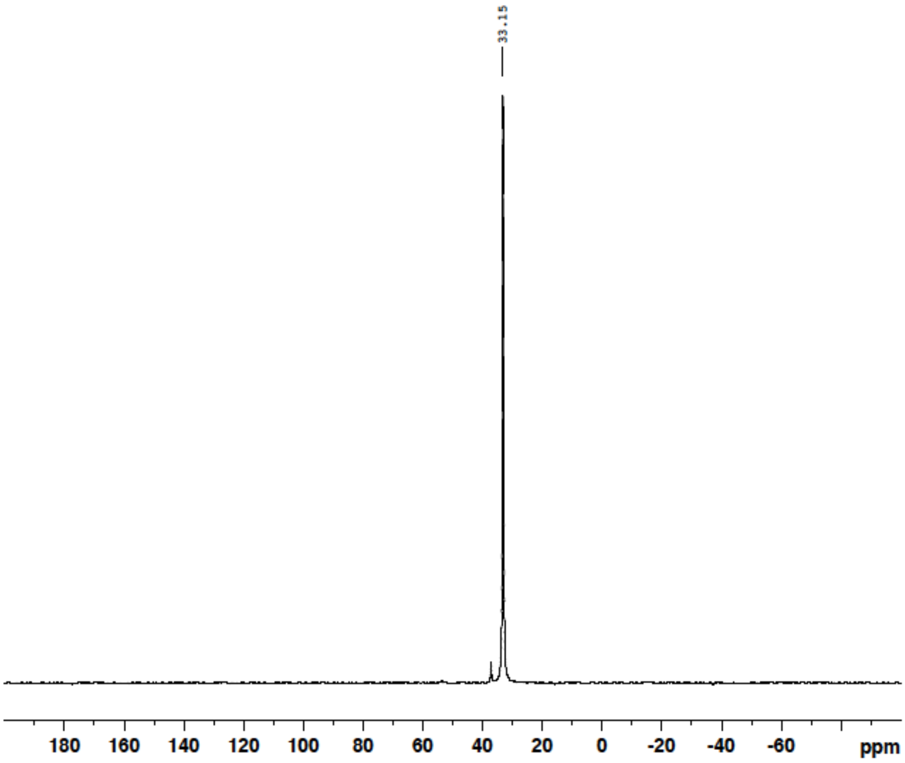

Figure ESI-8. ^{31}P NMR spectrum of mixture of DEGDBE and [$P_{4,4,4,8}$][BScB] ionic liquid with 0.60 mol kg^{-1} Li[BScB] salt.

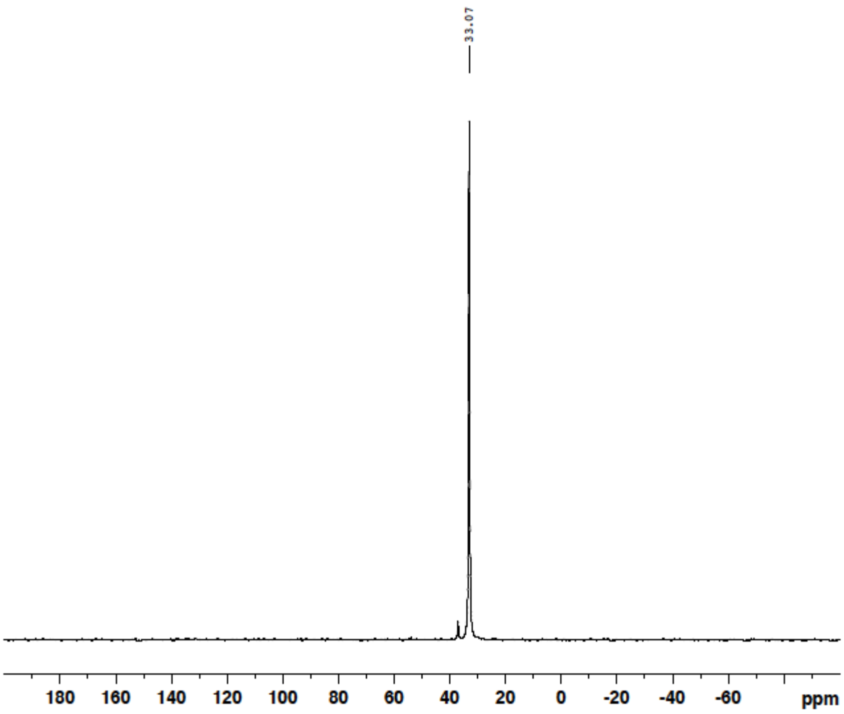

Figure ESI-9. ^{31}P NMR spectrum of mixture of DEGDBE and [$P_{4,4,4,8}$][BScB] ionic liquid with 1.0 mol kg^{-1} Li[BScB] salt.

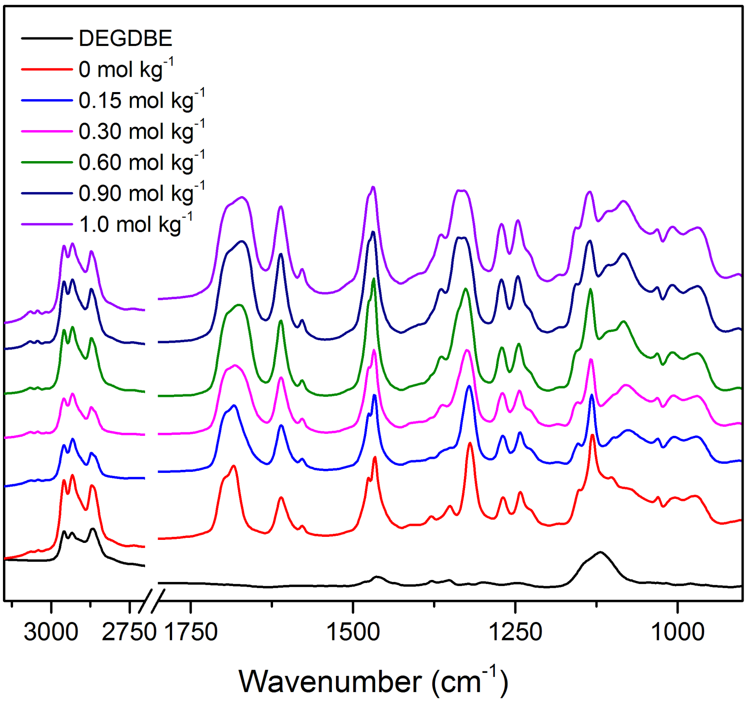

Figure ESI-10. FTIR spectra of bis(salicylato)borate ionic liquid based hybrid electrolytes with different concentrations of Li[BScB] salt.

Thermal Analysis

Thermogravimetric Analysis (TGA) was performed using a Perkin Elmer 8000 TGA apparatus. Temperature-ramped TGA experiments were carried out at a heating rate of 10 °C min. 2–3 mg of the electrolyte sample was used for each experiment. All the TGA experiments were performed under nitrogen gas as the inert carrier gas.

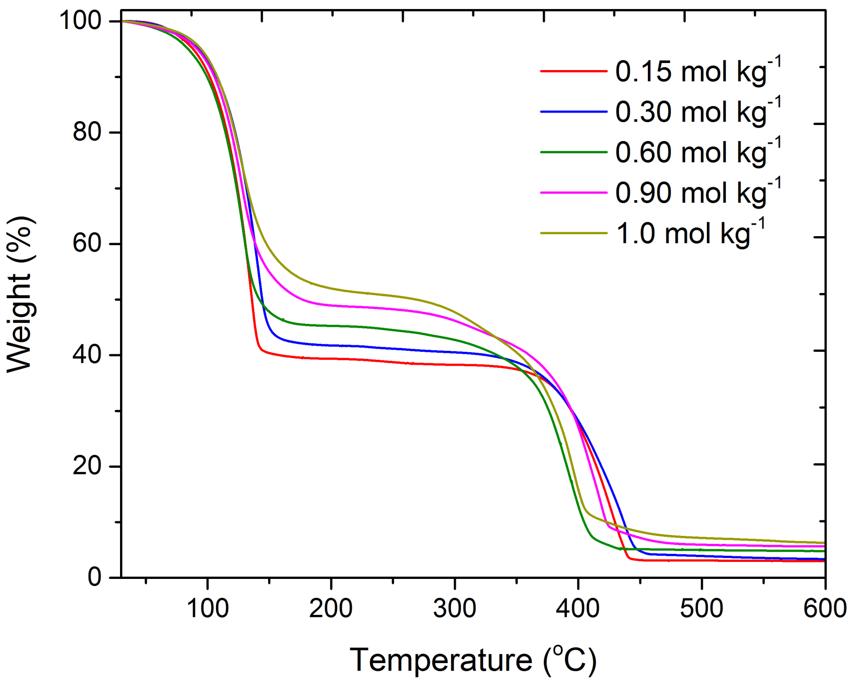

Figure ESI-11. TGA curves of bis(salicylato)borate ionic liquid based hybrid electrolytes with different concentrations of Li[BScB] salt.
